# The interaction of lipomatous hypertrophy of the interatrial septum with pericardial adipose tissue biomarkers by computed tomography

**DOI:** 10.1007/s00330-024-11061-3

**Published:** 2024-09-05

**Authors:** Pietro G. Lacaita, Thomas Senoner, Valentin Bilgeri, Stefan Rauch, Fabian Barbieri, Benedikt Kindl, Fabian Plank, Wolfgang Dichtl, Johannes Deeg, Gerlig Widmann, Gudrun M. Feuchtner

**Affiliations:** 1 Department of Radiology, Medical University Innsbruck, Innsbruck, Austria; 2 Department of Anaesthesiology and Intensive Care, Medical University Innsbruck, Innsbruck, Austria; 3 Department of Internal Medicine, Cardiology, Medical University Innsbruck, Innsbruck, Austria; 4 Department of Nuclear Medicine, Medical University Innsbruck, Innsbruck, Austria; 5 Department of Cardiology, Angiology and Intensive Care Medicine, Deutsches Herzzentrum der Charité, Berlin, Germany; 6 Department of Internal Medicine, Tyrol Clinicum Hall, Hall, Austria

**Keywords:** Computed tomography angiography, Adipose tissue, Biomarkers, Pericardium, Lipomatous hypertrophy of the interatrial septum

## Abstract

**Objective:**

Novel pericardial adipose tissue imaging biomarkers are currently under investigation for cardiovascular risk stratification. However, a specific compartment of the epicardial adipose tissue (EAT), lipomatous hypertrophy of the interatrial septum (LHIS), is included in the pericardial fat volume (PCFV) quantification software. Our aim was to evaluate LHIS by computed tomography angiography (CTA), to elaborate differences to other pericardial adipose tissue components (EAT) and paracardial adipose tissue (PAT), and to compare CT with [^18^F]FDG-PET.

**Materials and methods:**

Of 6983 patients screened who underwent coronary CTA for clinical indications, 190 patients with LHIS were finally included (age 62.8 years ± 9.6, 31.6% females, BMI 28.5 kg/cm^2^ ± 4.7) in our retrospective cohort study. CT images were quantified for LHIS, EAT, and PAT density (HU), and total PCFV, with and without LHIS, was calculated. CT was compared with [^18^F]FDG-PET if available.

**Results:**

CT-density of LHIS was higher (− 22.4 HU ± 22.8) than all other pericardial adipose tissue components: EAT right and left (97.4 HU ± 13 and − 95.1 HU ± 13) PAT right and left (− 107.5 HU ± 13.4 and − 106.3 HU ± 14.5) and PCFV density −83.3 HU ± 5.6 (*p* < 0.001). There was a mild association between LHIS and PAT right (Beta 0.338, *p* = 0.006, 95% CI: 0.098–577) and PAT left (Beta 0.249, *p* = 0.030; 95% CI: 0.024–0.474) but not EAT right (*p* = 0.325) and left (*p* = 0.351), and not with total PCFV density (*p* = 0.164). The segmented LHIS volume comprised 3.01% of the total PCFV, and 4.3% (range, 2.16–11.7%) in those with LHIS > 9 mm. [^18^F]FDG-PET: LHIS was tracer uptake positive in 83.3% (37.5%: mild and 45.8%: minimal) of 24 patients.

**Conclusions:**

LHIS is a distinct compartment of PCFV with higher density suggesting brown fat and has no consistent association with EAT, but rather with PAT.

**Clinical relevance statement:**

LHIS should be recognized as a distinct compartment of the EAT, when using EAT for cardiovascular risk stratification.

**Key Points:**

- *LHIS is currently included in EAT quantification software*.
- *LHIS density is relatively high, it is not associated with EAT, and has a high [*^*18*^*F]FDG-PET positive rate suggesting brown fat*.
- *LHIS is a distinct compartment of the EAT, and it may act differently as an imaging biomarker for cardiovascular risk stratification*.

## Introduction

Obesity is causing a high morbidity and mortality rate worldwide, and is an important cardiovascular risk factor. In the EU, 53% of adults are defined as overweight [1].

The body mass index (BMI) as a diagnostic criterion [1] for obesity has limited accuracy, due to a high proportion of “metabolic healthy obese” (MHO) [2], which are characterized by superior adipose tissue function [2]. However, even MHO may carry a residual risk of adverse outcomes [2]. Mounting evidence indicates that body fat distribution improves cardiovascular risk stratification [2]. Imaging biomarkers such as pericardial fat volume (PCFV) and density, and its components, the epicardial adipose tissue (EAT), paracardial adipose tissue (PAT), and pericoronary adipose tissue (PCAT), have shown incremental prognostic value for cardiovascular risk prediction [3–5] and could assist in tailoring novel therapeutic strategies for the treatment of obesity [1] such as glucagon-like peptide-1 receptor agonists (GLP-1RA) and other therapeutic agents.

Total PCFV quantified by computed tomography (CT) is a prognosticator for adverse outcomes in heart failure/cardiomyopathy [3] and coronary artery disease [4, 5]. PCFV consists of EAT and PAT [6]. PCAT is a distinct component of EAT, with an independent value for coronary artery disease risk stratification.

However, one specific PCFV compartment, lipomatous hypertrophy of the interatrial septum (LHIS)—has not yet been investigated extensively. LHIS is considered part of the EAT and is currently included in volume quantification by automated software [3]. LHIS has an estimated prevalence of 2–8% and varies in size [7].

LHIS may represent a distinct fat entity and has been reported to contain brown adipose tissue (BAT) [7, 8] with ^18^Fluordesoxy-d-glucose-positron emission tomography ([^18^F]FDG-PET) tracer uptake reported in a small series of 11 patients [7], but CT data are scarce [7, 9]. BAT has cardioprotective effects [10, 11], while white adipose tissue (WAT) attenuates the progression of coronary heart disease and heart failure.

Therefore, the purpose of our study was to quantify and characterize LHIS by computed tomography angiography (CTA), to elaborate on differences to total and other components of the PCFV (EAT, PCAT, and PAT), to define the LHIS % of PCFV and to determine the [^18^F]FDG-PET tracer uptake positive rate.

## Material and methods

### Study design and population

Patients who underwent electrocardiogram (ECG)-gated coronary or aortic CT- angiography for clinical indications were included in this retrospective cohort study. Institutional review board (IRB) approval was obtained, and written informed consent was waived by the IRB.

Inclusion criteria

- Patients referred because of suspected CAD and low-to-intermediate pre-test probability [12, 13] according to ESC 2019 and AHA 2021 chest pain guidelines, or other clinical indications (prior PCI/stent, CABG, EPS-planning, triple-rule out/pulmonary embolism ECG-gated CTA or others for coronary, and/or aortic ECG-gated CTA).
- Patients in whom LHIS was present on a clinical standardized radiology report from a small team of board-certified radiologists specialized in cardiac CT imaging (> 10 years of experience)

## CT

### Calcium score

A non-contrast ECG-gated CT scan with standardized scan parameters (detector collimation 2 × 64 × 0.6 mm; 120 kV; image reconstruction 3 mm slice width, increment 1.5), and prospective ECG-triggering in high-pitch (dual source) mode and into diastolic phase was performed. The Agatston units (AU) [14] of all coronary arteries were calculated with automated software (CT Cardiac, SyngoVIA, Siemens Healthineers).

### Coronary CTA

Coronary CTA was performed by using 128-slice dual-source CT (Definition FLASH or DRIVE, Siemens Healthineers) with a detector collimation of 2 × 64 × 0.6 mm and a rotation time of 0.28 s, acquiring 128-slices with z-flying spot. Scans were triggered into the arterial phase using bolus tracking (threshold of 100 Hounsfield units (HU), ascending aorta). An iodine contrast agent was injected (Iopromide, Ultravist 370™, Bayer Healthcare*)* into an antecubital vein using an automated injector. The contrast media volume was calculated using a standardized regimen, based on the patient´s body weight. Contrast medial volume and flow rate (4 mL/s, 5 mL/s, or 6 mL/s) were adjusted according to the patient’s weight. The contrast media volume ranged from 60 mL up to 110 mL. Prospective ECG-triggering (< 65 bpm) or retrospective ECG-gating (> 65 bpm or arrhythmia) was performed depending on heart rate. Tube voltage ranged from 80 kV to 140 kV, and was adjusted to the patients’ body mass and dimensions using an automated tool (CARE kV^TM^, Siemens Healthineers). Similarly, tube currents (mAs) were adapted automatically (CARE Dose4D^TM^, Siemens Healthineers).

Patient preparation: Patients received betablockers if the baseline heart rate was above 80 bpm, and optionally, if above 65 bpm. Five mililitre Metoprolol (Beloc^TM^) was injected intravenously into an antecubital vein, and repeated after 5 min, if necessary. Oral premedication with betablockers was recommended to referring physicians 1 h prior to CT if possible.

Axial thin slice images at best diastolic and systolic phase were reconstructed with 0.75 mm slice width (increment, 0.4) and transferred to a 3D-postprocessing software (CT Cardiac; SyngoVIA, Siemens Healthineers).

**Fig. 1:**
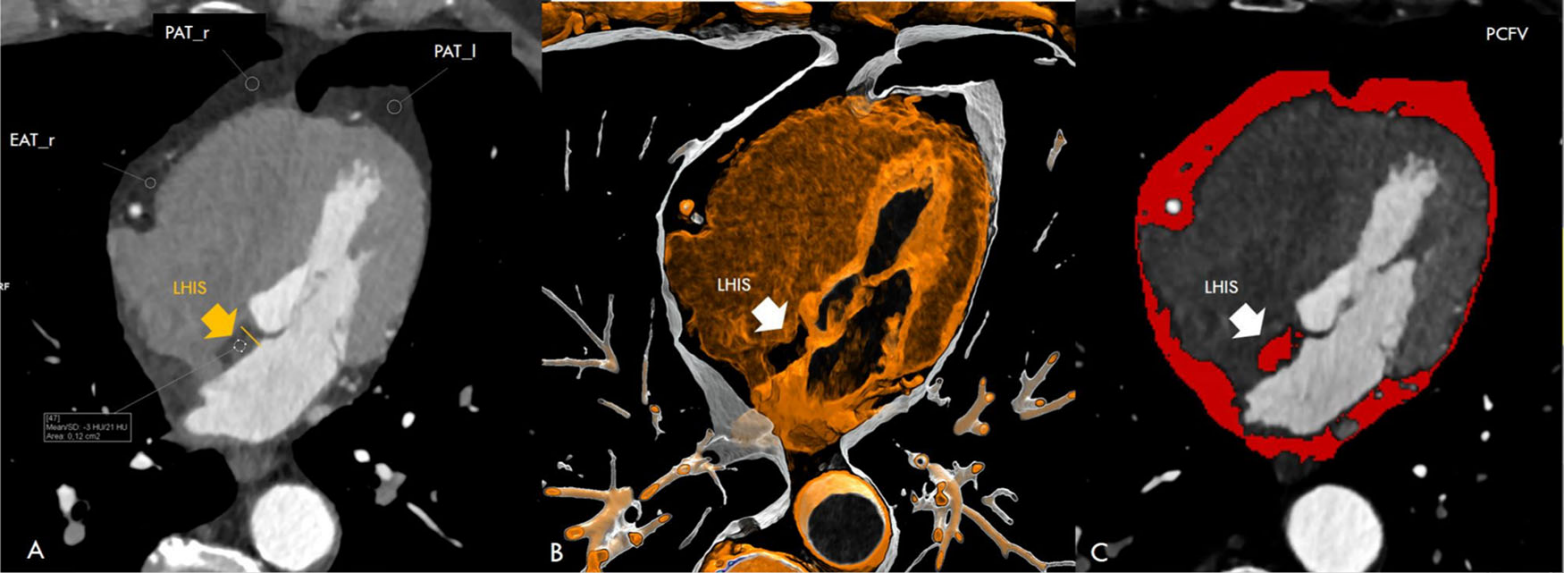
Quantification of LHIS density and size (**A**, **B**) and total PCFV by CT (**C**, by using SyngoVIA Frontiers, cardiovascular risk assessment) prior to editing and exclusion of LHIS in 66-years-old male (BMI 28.4 kg/cm^2^) male referred to coronary CTA. Axial CT images show LHIS (arrow), quantification of LHIS, EAT, and PAT density, and measurement of LHIS width (orange line, **A**)

Coronal multiplanar reformations were used to quantify the following PCFV compartments (Fig. 1):

LHIS was defined as mass-like lipomatous tissue infiltration of the entire interatrial septum from cranial to caudal, sparring the fossa ovalis, with a “dumb-bell” configuration.

1. The maximal width was identified on axial images and measured with a digital caliper. The maximal length was defined as the distance from the anterior to the posterior borders.
2. CT density: a circular region of interest (ROI) was placed into the area of maximal LHIS width.

### PCAT

PCAT consists of adipose tissue located within the EAT, defined as any adipose tissue within a radial distance of less than 5 mm from the external vessel wall equivalent to the diameter of the coronary vessel [15]. A round-shaped ROI was placed within less than a 5 mm radius of the proximal (or mid) RCA, and anteriorly of the LCA/proximal LAD bifurcation into fatty tissue.

### EAT

EAT was defined as the adipose tissue located between the myocardium and the visceral layer of the pericardium [16]. For quantification of CT density, a round-shaped ROI with approximately 20 mm^2^ size was placed into the right EAT compartment, if possible, at the mid-section (of the cranio-caudal heart scan range)—lateral of the right ventricle. If there was no EAT within the mid-section present, the right EAT was quantified more cranially or caudally. Then, left EAT was measured at the most appropriate site (if possible, mid-section) of the LV, if possible, at the same slice level of the right, or if not, at the closest possible distal or proximal slice level. The left ROI was drawn with the same circular dimensions as the right, with an average size of 20 mm^2^, into an area of homogenous adipose tissue, and by sparing small vessels or other structures such as fibrous bands.

### PAT

PAT is anatomically located external to the parietal pericardium. PAT was measured with a circular ROI of approximately the same size as used for EAT, and placed into both left and right PAT compartments—as described above for EAT, respectively.

### Reference values

Subcutaneous (SC) fat density (HU) was measured. The image noise (standard deviations (SD) of HU) of the LHIS was recorded as an image quality parameter.

When placing the ROI, care was taken to avoid artifacts (such as motion blurring) or dense structures such as small feeding vessels or fibrous bands.

All CT measurements listed above were performed by experienced observers with more than 3 years of radiology training. The coronary artery disease results from standardized reporting of the CTA according to CADRADS^TM^ [17]—including stenosis severity classification, were also transferred.

### PCFV quantification (Fig. 1c)

PCFV refers to the adipose tissue consisting of both EAT and PAT according to Bertaso et al [6].

A client-server-based software (SyngoVIA Frontiers, Cardiovascular Risk Assessment, Siemens Healthineers) was used to segment and quantify PCFV. After adjusting the threshold to −30 HU (lower) and −190 HU (upper) limits according to current standardized recommendations [18], the total PCFV and density were recorded. Then, the PCFV borders were edited manually: the LHIS volume was excluded, and the PCFV was re-segmented and calculated. The total volume of LHIS was calculated by subtracting PCFV after re-segmentation from prior PCFV and presented as % of the total PCFV.

### [^18^F]FDG-PET

A combined [^18^F]FDG-PET/CT scanner (GE Discovery 690, General Electric) was used. Fused images with a 5.5 mm average slice thickness were reconstructed. Patients were advised to fast at least 6 h prior to the exam. Fifteen to twenty mCi of [^18^F]FDG-tracer was administered intravenously as a bolus, and static images were obtained 60 min later.

[^18^F]FDG tracer uptake rate was scored visually by a nuclear medicine board-certified observer (S.R.) and maximal SUV uptake of the LHIS was quantified, the SUV of the left ventricular myocardium, the liver, and SC fat as reference using a specific commercial software (AW-Server 3.2, Extension 4.8, General Electric). Increased tracer uptake was defined as less, equal, or more than the background mediastinal activity and scored as positive (1 = mild, 2 = minimal, questionably borderline positive) or negative (0). Major traditional cardiovascular risk factors (CVRF) were collected and defined according to standardized European Society of Cardiology criteria: arterial hypertension (systolic blood pressure (BP) > 140 mmHg or diastolic BP > 90 mmHg), dyslipidemia, positive family history (myocardial infarction or sudden cardiac death in an immediate male relative < 55 years or female < 65 years), smoker (active: current or quit less than 6 months before CTA examination and former), and diabetes [19–21].

### Statistical analysis

Statistical analysis was performed using SPSS™ software (V29.0, SPSS Inc.). Quantitative variables are expressed as means ± SD or as median (IQR), and categorical variables as absolute values and percentages. The normal distribution of data was tested with a histogram and the Kolmogorov test.

Spearman correlation coefficient was determined for the correlation of LHIS tissue CT density with other PCFV tissue components (PAT, EAT, and PCAT).

The *t*-test was applied to test for differences in tissue CT-densities (HU) of LHIS and other PCFV components (PAT, EAT, and PCAT), and linear regression analysis for associations among them; as well for tube kV with the PCFV compartments EAT and LHIS. Mann–Whitney *U*-test was applied to test for gender differences and for differences in LHIS density (HU) between obese and non-obese (BMI cutoffs of > 25 kg/m^2^ for overweight and 30 kg/m^2^ for obese). A two-sided *p* value of ≤ 0.05 was defined as significant.

## Results

**Fig. 2:**
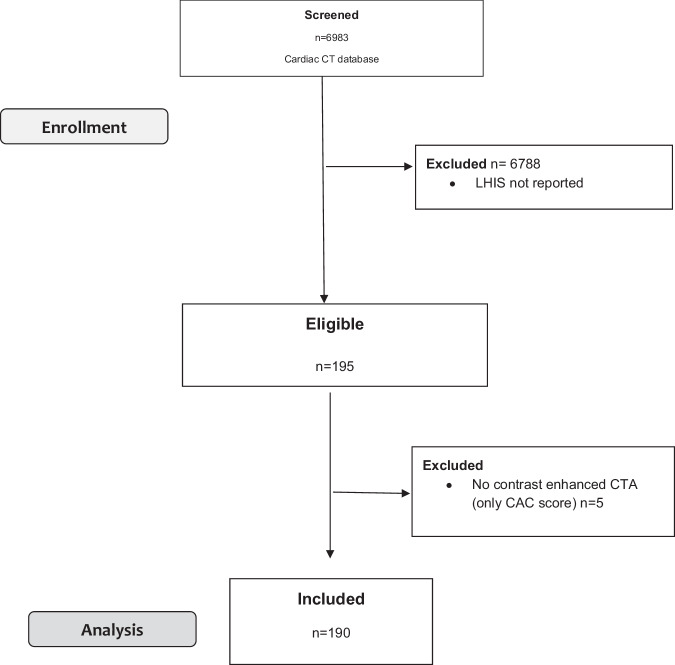
Patient flow chart

The study cohort profile is shown in Table 1. A CONSORT diagram for patient enrollment, inclusion, and exclusion is shown in Fig. 2. Out of 6983 patients screened who underwent cardiac CTA for clinical indications, 195 patients who had LHIS reported were enrolled (prevalence, 2.8%).

**Table 1:** Study cohort: patient profile (*n* = 190)

Age (y)	62.9 ± 9.6
Females	60 (31.6%)
BMI (kg/m^2^)	28.6 ± 4.7
Overweight or obese (BMI > 25 kg/m^2^) Overweight (> 25–30 kg/m^2^) Obese (BMI > 30 kg/m^2^)	146 (76.8%) 78 (41.05%) 68 (35.7%)
Smoking	70 (36.8%)
Arterial hypertension	79 (41.6%)
Positive family history	39 (20.5%)
Dyslipidemia	87 (45.8%)
Diabetes	29 (15%)
**Coronary CTA results**	***N = 163***
CAC score	254.8 AU (range, 0–3312.1 AU)
CADRADS	
0	31 (19%)
1	16 (9.8%)
2	48 (29.4%)
3	21 (12.9%)
4	46 (28.2%)
HRP	31 (19%)
Prior stent or CABG, other indications (EPS planning, TRO/PE)	*N* = 22, *N* = 5

Parametric variables are expressed as means ± SD, categorical variables as absolute values (*N*), and percentages (%)

*Y* years, *BMI* body mass index, *FH* family history, *CAGB* coronary artery bypass grafting, *CAC* coronary artery calcium, *CADRADS* coronary artery disease radiological reporting system (stenosis severity), *HRP* high-risk-plaque, *TRO* triple rule out, *PE* pulmonary embolism, *CTA* computed tomography angiography, *AU* Agatston units, *EPS* electrophysiological procedure planning (e.g., pulmonary vein ablation)

Five patients were excluded because only a non-enhanced CT scan for calcium scoring was performed, and CTA was deferred for contraindications. Finally, 190 patients were included in the study. Of 190 patients, 163 were referred for evaluation of suspected coronary artery disease, 22 due to prior CABG or stent follow-up, and 5 for other indications for ECG-gated coronary or thoracic/aortic CTA (left atrial ablation planning, Triple-rule-out or transcatheter aortic valve intervention planning). Major CVRF and CTA results (CAC score, CADRADS, and high-risk plaque prevalence) from standardized clinical CTA reporting are presented in Table 1.

Quantitative CT-analysis data for LHIS is shown in Table 2. LHIS dimensions were: width, mean 7.3 mm ± 2.5 (range, 3.2–28 mm), and length, mean 32.5 mm ± 7.3 (range, 15–87 mm).

**Table 2:** Quantification of LHIS, total PCFV and density, and its compartments by CTA

		Mean ± SD	Range
LHIS			
Size	Width (mm)	7.3 ± 2.5	3.2–28
	Length (mm)	32.5 ± 7.3	15–87
Density (HU)	Neg HU (−), Pos HU (+)	−22.4 ± 22.8 166 (87.3%), 24 (12.6%)	−85, −45
PCAT (HU)	Right	−95.3 ± 15	−141 to −40
	Left	−97.9 ± 17	−142 to −46
EAT (HU)	Right	−97.4 ± 13	−133 to −50
	Left	−95.1 ± 13	−149 to −55
PAT (HU)	Right	−107.5 ± 13.4	−143 to −60
	Left	−106.3 ± 14.5	−136 to −64
Total PCFV	mL	268.4 ± 107	98.6 to 644.56
PCFV density	HU	−83.5 ± 5.8	−66 to −102
SC fat (HU)	Left	−107.09 ± 12.6	−139 to −55
Image noise	SD of HU	19.05 ± 8.2	1 to 51

*LHIS* lipomatous hypertrophy of the interatrial septum, *PCAT* pericoronary adipose tissue, *EAT* epicardial adipose tissue, *PAT* paracardial adipose tissue, *PCFV* pericardial fat volume, *SC* subcutaneous fat, *HU* Hounsfield units, *mm* millimeter, *SD* standard deviations

**Fig. 3:**
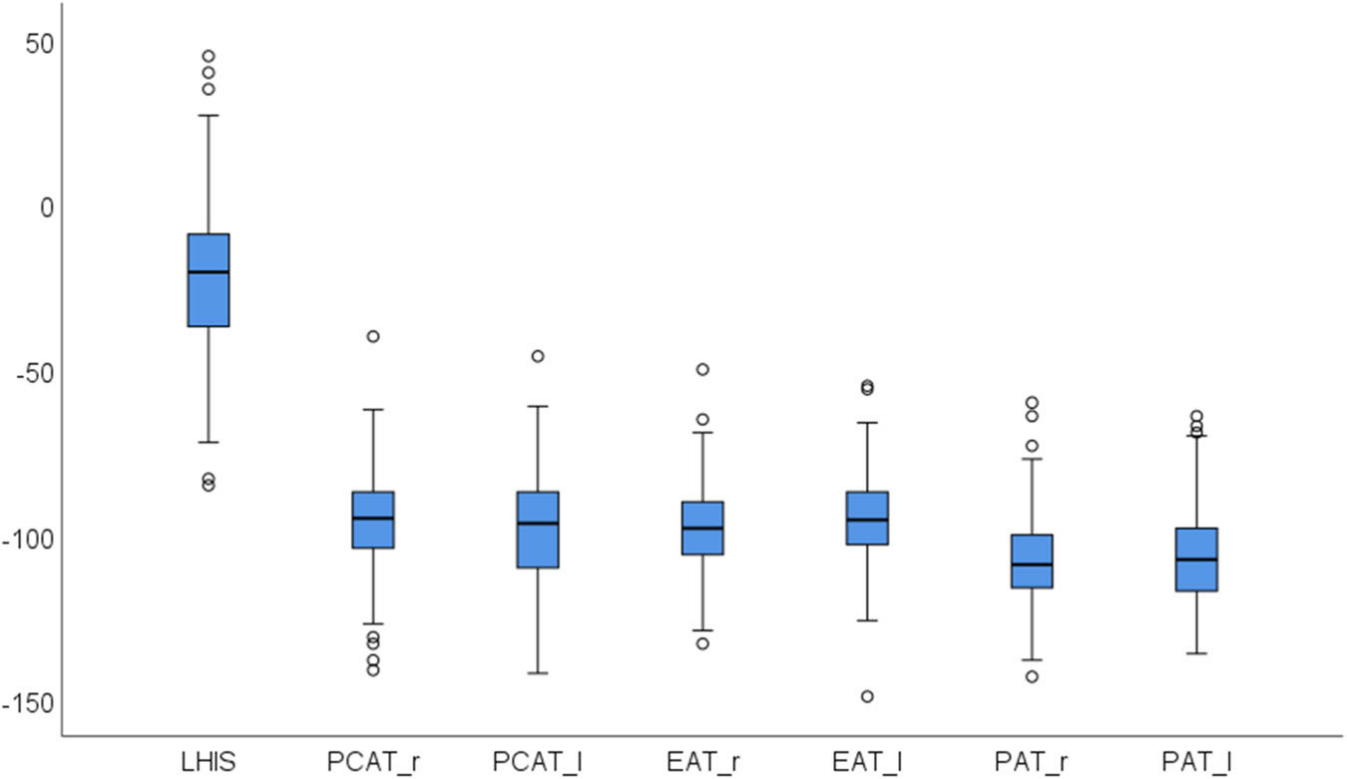
Boxplot: CT density (HU) of LHIS was significantly (*p* < 0.001) higher as compared to other PCFV components (PCAT, EAT, and PAT; r = right and l = left). *y*-axis: CT density (HU)

CT density of LHIS was significantly higher (−22.4 HU ± 22.8) than all other pericardial adipose tissue components (PCAT right: −95.3 HU ± 15, PCAT left: −97.9 HU ± 17, EAT right: −97.4 HU ± 13 and EAT left: −95.1 HU ± 13, PAT right: −107.5 HU ± 13.4 and PAT left: −106.3 HU ± 14.5; *p* < 0.001 for all) (Fig. 3), and total PCFV density (− 83.3 HU ± 5.6; *p* < 0.001).

There was a weak correlation of LHIS density with PAT left and right (*r* = 0.145, *p* = 0.046 and *r* = 0.179, *p* = 0.013), but no correlation of LHIS density with EAT, PCAT, and total PCFV density (*r* = 0.050, *p* = 0.590).

**Fig. 4:**
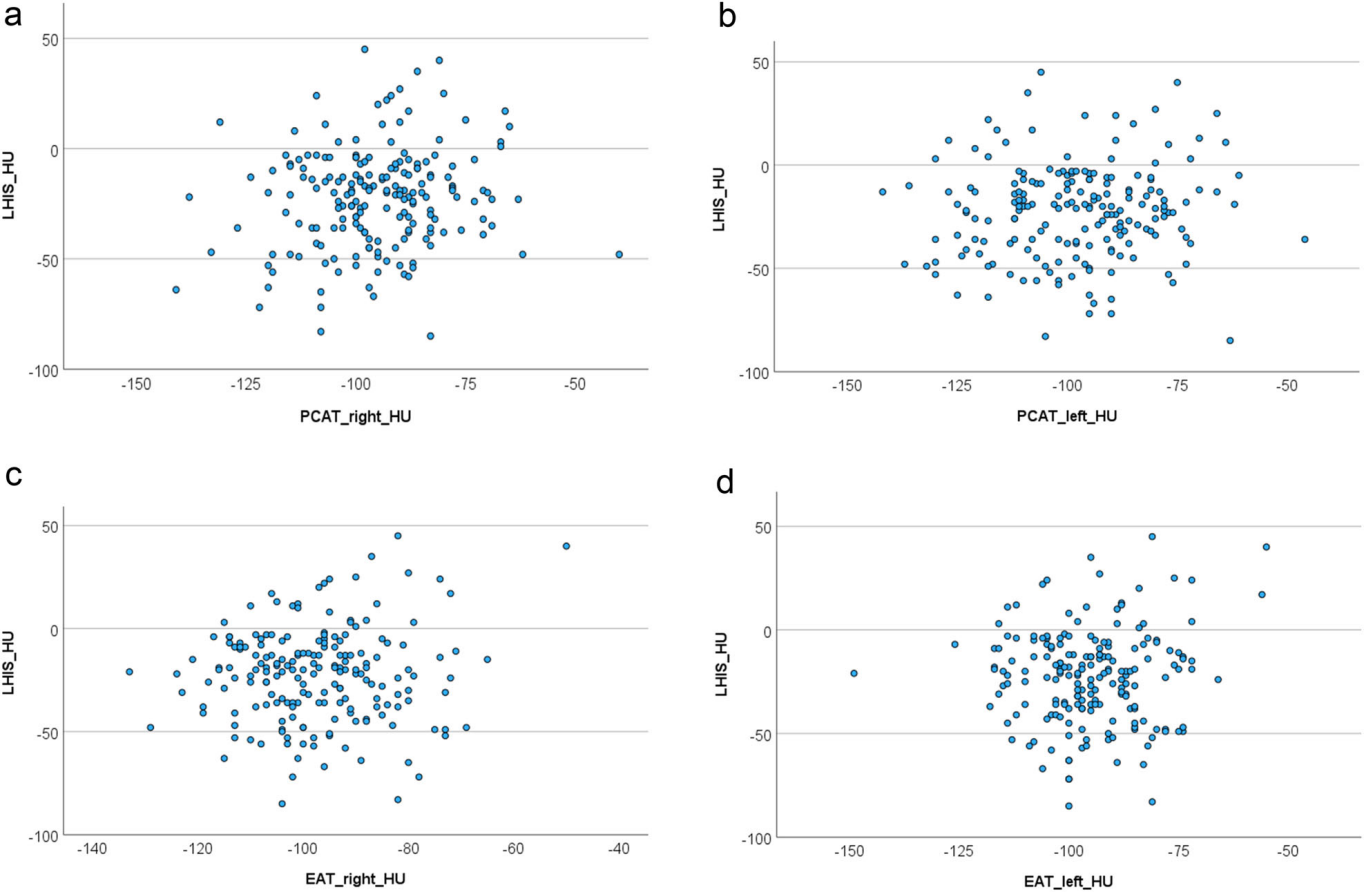
Linear regression analysis. **a** CT density (HU) of LHIS compared to PCAT right showed a significant association (Beta 0.227, 95% CI: 0.010–0.445, *p* = 0.041). **b** CT density (HU) of LHIS compared to pericoronary artery tissue (PCAT) left did not show a correlation (Beta 0.105, 95% CI: −0.087–0.297, *p* = 0.281). **c** CT density (HU) of LHIS was not associated with EAT right (Beta 0.128, 95% CI: −0.127–0.383, *p* = 0.325). **d** CT density (HU) of LHIS was not associated with EAT left (Beta 0.121, 95% CI: −0.134–0.377, *p* = 0.351) was not associated

**Fig. 5:**
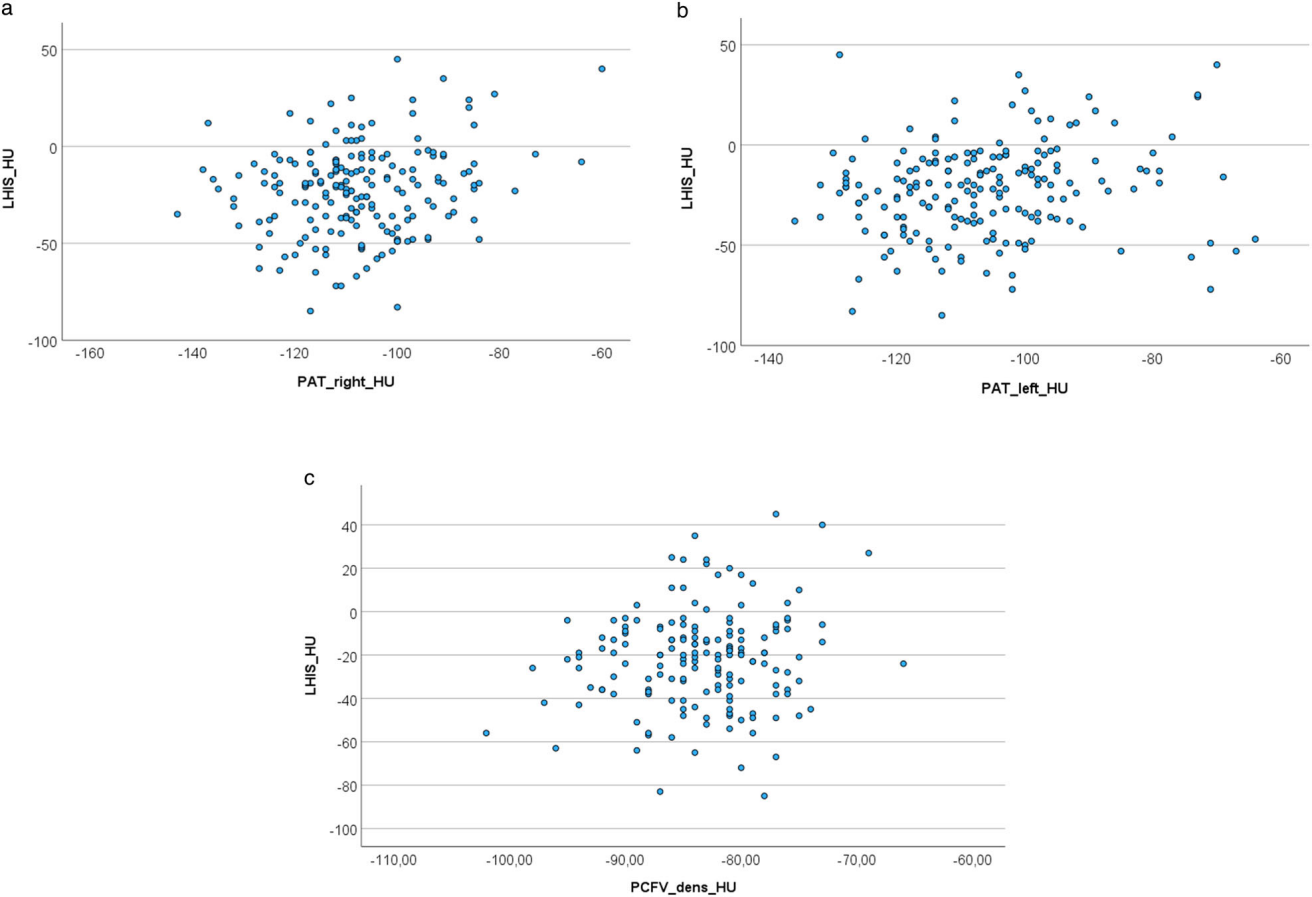
Linear regression analysis. **a** CT density (HU) of LHIS compared to PAT right showed a significant association (Beta 0.338, 95% CI: 0.098–0.577, *p* = 0.006). **b** CT density (HU) of LHIS compared to PAT left were correlated (Beta 0.249, 95% CI: 0.024–0.474; *p* = 0.030;). **c** CT density (HU) of LHIS compared to total PCFV density showed no correlation (Beta 0.545, *p* = 0.076, 95% CI: −0.058–1.148)

Linear regression analysis (Figs. 4 and 5). There was an association between the CT density of LHIS and PAT right (Beta 0.338, *p* = 0.006; 95% CI: 0.098–0.577) and PAT left (Beta 0.249, *p* = 0.030; 95% CI: 0.024–0.474) but not EAT right (*p* = 0.325) and left (*p* = 0.351). There was no association between CT density of LHIS and PCAT left (*p* = 0.281), and a borderline significant association with PCAT right (Beta 0.227, *p* = 0.041; 95% CI: 0.010–0.445) and no association with total PCFV-density (Beta 0.568, *p* = 0.164; 95% CI: −0.236–1.37).

### BMI and age

There was no difference in the LHIS density between obese, overweight, and non-obese patients, for both BMI cut-offs of 25 kg/m^2^ (22.5 ± 1.8 vs 25.5 ± 3.8, *p* = 0.758) and 30 kg/m^2^ (22.6 HU ± 21.4 vs 22.9 HU ± 32.7, *p* = 0.706). There was a weak but significant correlation between age and LHIS density (*r* = − 0.160, *p* = 0.027).

### Gender differences

There was no difference in the CT density of LHIS between males and females (*p* = 0.196).

### PCFV before and after the exclusion of LHIS volume

Total PCFV was mean 268.4 mL ± 107 SD (range, 98.5–644.6 mL). Segmented LHIS volume was mean 8.11 mL ± 5.1 in a subset of 128 patients with LHIS ≥ 5 mm, resulting in a proportion of mean 3.01% ± 1.4 SD of total PCFV. In large LHIS > 9 mm width (23 patients), LHIS volume was mean 12.8 mL ± 6.8 SD (range, 4.4–27.6 mL) and 4.3% ± 1.96 (range, 2.16–11.7%) of total PCFV.

### Coronary artery disease profile by CTA

CAC and coronary stenosis severity by CADRADS compared to LHIS and other PCFV components. There was a non-significant trend (*p* = 0.09) with an inverse relationship between CAC score and LHIS density (*r* = −0.138), while all other PCFV components were not correlated. There was no significant correlation of LHIS density (HU) with CADRADS (*p* = − 0.124, *p* = 0.117).

### [^18^F]FDG-PET

**Fig. 6:**
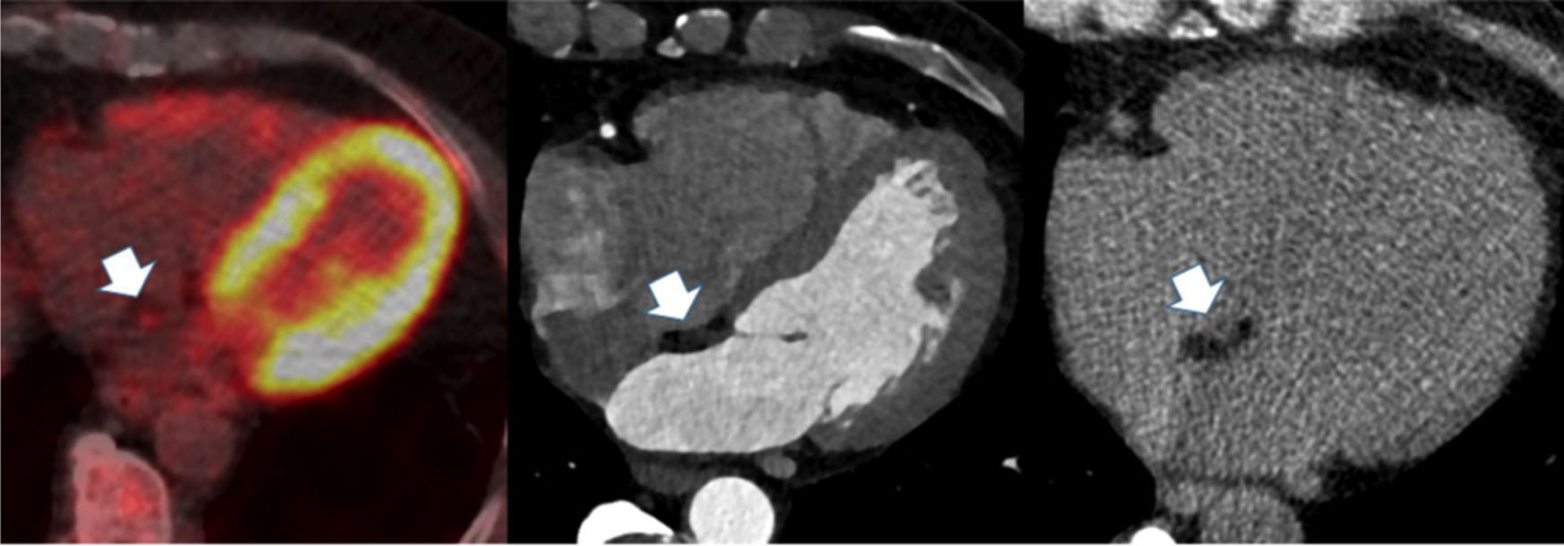
A 64-year-old male (BMI, 24.2 kg/cm^2^) with high LHIS density (mean + 24 HU). Mild [^18^F]FDG tracer uptake (left panel) central. White arrow pointing at LHIS on coronary CTA (mid panel) and unenhanced CAC score (right panel)

In 24 patients who underwent PET, LHIS was tracer negative in 4 (16.7%) and positive in 20 (83.3%). Nine of 24 (37.5%) were scored as mild, and 11 (45.8%) as minimally positive (Fig. 6). SUV uptake of LHIS was mean 2.23 ± 0.87 (range, 1.2–4.75). SUV in the liver was mean 5.13 ± 1.8 (range, 2.97–9.63), the LV myocardium 6.64 ± 5.5 (range, 2.18–23.9), and SC fat 0.822 ± 0.27 (range, 0.44–1.6). The ratio LHIS/liver was mean 0.456 (range, 0.18–0.79). [^18^F]FDG-PET was performed after CTA in 16 (66.7%) patients, and before in 8 (33.3%). The time interval was less than 2 up to maximal 3 years in the majority (91.7%) of patients.

Tube voltage was 100 kV or 120 kV in the majority (87.9%), 80 kV or 90 kV in ten patients (5.3%), and 130 kV or 140 kV in 13 patients (6.8%).

The mean dose length product (DLP) was 647.6 ± 344 SD mGy⋅cm and the computed tomography dose index (CTDI) volume was 40.68 ± 23.4 SD mGy.

The influence of tube voltage on EAT and LHIS attenuation: There was no association between LHIS density (HU) and tube voltage (kV) on univariate linear regression analysis (*p* = 0.406). There was no association of tube kV on multivariate linear regression including the covariates: LHIS, EAT right and left density HU with *p* = 0.400, *p* = 0.259, and *p* = 0.628, respectively.

## Discussion

Our study shows that the CT density of LHIS is different and markedly higher than all other PCFV tissue components (EAT, PAT, and PCAT). LHIS is a benign tumor-like lipomatous infiltration of the interatrial septum, that has been surmised to arise from EAT [1]. However, for the first time, our study revealed that LHIS density is distinct from EAT, and rather associated with PAT.

The two major PCFV adipose tissue components are EAT and PAT [6]. PCFV consists of a visceral fat type, and two major compartments: EAT (located underneath the pericardial layer) and PAT (outside the pericardium)—according to the originally proposed universal standardized terminology introduced in 2013 by Bertaso et al [6]. However, large prospective studies later revealed that only EAT volume [3–5] is associated with adverse cardiovascular outcomes and elevated biomarkers of inflammation [22], while PAT is considered a “benign” fat potentially containing brown adipose tissue [3]. Nerlekar et al [23] monitored EAT volume and density changes over time and concluded that EAT is an independent parameter with temporal changes. Accordingly, both EAT density and volume could be used as treatment response indicators for novel therapeutic agents in obesity.

Total EAT volume is a valuable prognosticator of CV risk, and consumes the major part of the pericardial fat. We found that LHIS with > 9 mm of width involved an average of 4.3% of the total PCFV. Of note, threshold adaptations of upper and lower HU borders were not performed in order to adhere to standardized general recommendations (− 190 HU and − 30 HU) for PCFV segmentation [18]. Because CT attenuation higher than −30 HU was frequently observed within LHIS, the percentage of absolute effective LHIS volume may be even higher.

The true nature of LHIS tissue has not been extensively investigated in systematic studies. Only two studies so far have quantified LHIS by CT [7, 9] by using older scanner generations than the 128-slice dual-source CT scanner applied in our study, and only one study [7] performed a correlation with [^18^F]FDG-PET in a subset analysis including 11 patients, in which 81.8% were PET positive—similar to our study. A few case reports [24–27] have shown [^18^F]FDG-PET-positive LHIS, and one showed temporal changes in tracer uptake [27]. LHIS has been presumed to contain BAT, due to FDG-tracer uptake. Indeed, the higher density of LHIS found in our study is suggestive of BAT. Subscapular BAT has shown densities of around—50 HU up to −90 HU [28]. The tissue density of LHIS quantified in our study was even higher, which may be explained by the different location of LHIS (intrathoracic) as compared to the scapula, or by more fibrous tissue within the interatrial septum. We report the largest series of patients with [^18^F]FDG-PET correlations so far. [^18^F]FDG-tracer uptake was minimal or mild in our patient population, and only slightly higher compared to the study by Fan et al [7]. These results are suggestive of brown adipose tissue (BAT) infiltration rather than white adipose tissue (WAT). WAT does not show FDG-tracer uptake due to a lack of metabolic active tissue components.

The CT density of adipose tissue is dependent on the proportion of fibrous vs lipid droplets. White adipose tissue shows lower HU because it contains larger droplets and has a higher lipid content. Lower EAT density (HU) has shown an even stronger association with major adverse cardiovascular events (MACE) than EAT volume and even the coronary calcium score [5].

Lastly, we discovered a marginally significant and weak association between LHIS and the CT density of right PCAT and LHIS. PCAT is a distinct sub-type of EAT that is linked to pericoronary inflammation, high-risk plaques, and an increased risk of cardiovascular events. Specifically, right PCAT density is associated with the occurrence of myocardial ischemia [29], while PCAT volume, EAT, and PAT are not. The reason remains uncertain. In a meta-analysis (20 studies, 7797 patients) higher PCAT suggestive of pericoronary inflammation was found in unstable plaques compared with stable plaques and offered incremental prognostic value for MACE prognostication [30], with promising performance when integrated into CV event prediction models [31].

Age was also associated with an elevated LHIS lipid component with lower CT attenuation, similar to other studies [8], but BMI was not. These findings support the BMI’s limited ability to identify high-risk obese individuals with dysfunctional adipose tissue.

The mean size of LHIS in our cohort was rather small, but similar to the landmark study by Fan et al [7]. Of note, LHIS larger than 2 cm in size may absorb a greater percentage % of the total PCFV.

### Limitations

We acknowledge the retrospective study design with all its potential biases. The mean BMI in our study was high, and the majority of patients were defined as overweight or obese, however, non-obese patients were also included. Tube voltage may have a slight influence on CT-density quantification; this effect was very small and did not cause a significant impact in a prior investigation due to the compensatory effect of BMI [32], and similarly, we found no association. Further, we acknowledge that underreporting of LHIS prevalence may affect the true prevalence, due to the benign nature of the lesion.

Finally, the radiation dose was rather high in our cohort, explained by the higher BMI and the higher rate of obesity in patients with LHIS compared to normal patients with low-to-intermediate pre-test probability referred to CTA, and the rather high prevalence of COPD patients with high heart rates requiring retrospective spiral ECG-gated CTA.

## Conclusion

LHIS density is higher than total PCFV density and its compartments (EAT, PAT, and PCAT) indicating a distinct adipose tissue type (for example, BAT or more fibrous-dense adipose tissue with smaller droplets).

Rather than EAT and PCAT, LHIS’s CT density is more closely linked to PAT. LHIS volume, in LHIS > 9 mm size, comprises 4.3% of the entire PCFV. The high (83.3%) [^18^F]FDG-PET positive rate may be indicative of BAT, with tracer uptake ranging from minimal to mild.

### Clinical perspective

LHIS should be recognized as a distinct compartment of EAT when using EAT as a prognosticator for adverse cardiovascular outcomes. Possibly, LHIS might be excluded due to its high probability of containing brown adipose tissue, a positive biomarker for metabolic health—similar to PAT.

However, further systematic research in larger sample sizes is required to gain more insights into the role of LHIS for risk stratification in obese and its interaction with other imaging and serum biomarkers.

In light of the “obesity pandemic”, and the fact that imaging biomarkers add incremental value to cardiovascular risk stratification, our study adds novel insights into pericardial adipose tissue biomarkers predicting CV risk; this could assist in tailoring novel therapies such as GPL-1 RA. Epicardial fat volume quantification might also be used to monitor treatment response over time to these therapies.

## Abbreviations

[^18^F]FDG-PET: ^18^Fluordesoxy-d-glucose-positron emission tomography
BMI: Body mass index
CT: Computed tomography
CTA: Computed tomography angiography
EAT: Epicardial adipose tissue
ECG: Electrocardiogramm
HU: Hounsfield units
LHIS: Lipomatous hypertrophy of the interatrial septum
PAT: Paracardial adipose tissue
PCAT: Pericoronary adipose tissue
PCFV: Pericardial fat volume
SD: Standard deviations
